# Association between Delivery during Off-Hours and the Risk of Severe Maternal Morbidity: A Nationwide Population-Based Cohort Study

**DOI:** 10.3390/jcm12216818

**Published:** 2023-10-28

**Authors:** Il Yun, Eun-Cheol Park, Jin Young Nam

**Affiliations:** 1 Department of Public Health, Graduate School, Yonsei University, Seoul 03722, Republic of Korea; ilyun94@yuhs.ac; 2 Institute of Health Services Research, Yonsei University, Seoul 03722, Republic of Korea; ecpark@yuhs.ac; 3 Department of Preventive Medicine, Yonsei University College of Medicine, Seoul 03722, Republic of Korea; 4 Department of Healthcare Management, Eulji University, Seongnam 13135, Republic of Korea

**Keywords:** severe maternal morbidity, maternal health, off-hour delivery, nighttime delivery

## Abstract

This study evaluated the association between off-hour deliveries and the risk of severe maternal morbidity (SMM). Data regarding Korean deliveries between 2005 and 2019 obtained from the National Health Insurance Service were used. SMM was evaluated using an algorithm developed by the United States Centers for Disease Control and Prevention. Modified Poisson regression analyses were conducted to investigate the association between off-hour deliveries and SMM, with stratification by hospital region and the number of beds. Approximately 32.7% of the 3,076,448 nulliparous women in this study delivered during off-hours, including 2.6% who experienced SMM. Patients who delivered at night had the highest risk of SMM (weekday nights, adjusted risk ratio (aRR): 1.41, 95% confidence interval (CI): 1.38–1.44; weekend nights, OR: 1.40, 95% CI: 1.34–1.46). The SMM of night deliveries was higher at hospitals located in small cities and those with 100–499 beds (weekend night: small cities, aRR: 1.49, 95% CI: 1.40–1.59; 100–499 beds, aRR: 1.83, 95% CI: 1.67–2.01; weekday night: small cities, aRR: 1.46, 95% CI: 1.42–1.51; 100–499 beds, aRR: 1.70, 95% CI: 1.62–1.79). Therefore, nighttime deliveries are associated with a higher risk of SMM, especially at hospitals located in small cities and those with 100–499 beds.

## 1. Introduction

Maternal mortality has been a global priority for decades, as evidenced by Millennium Development Goals [1] and Sustainable Development Goals [2] adopted by the United Nations. While the maternal mortality rate (MMR) has decreased significantly over the past two decades due to global efforts, poor maternal health remains a burden in underdeveloped, developing, and developed countries [3]. Korea’s MMR was 7.8 per 100,000 live births in 2017, and increased to 11.8 in 2020, which exceeded the average MMR of 10.9 for Organization Economic Cooperation and Development countries [4].

Severe maternal morbidity (SMM) is a more useful indicator than MMR for assessing the quality of obstetric care [5]. SMM is characterized by adverse outcomes of labor and labor processes that have significant short- or long-term consequences for women’s health [6]. Risk factors affecting the occurrence of SMM have been identified in several previous studies. Older maternal age [7], multiple births [7], obstetric comorbidities [8], cesarean sections [9], and preterm births [10] increase the risk of SMM. In addition, the patient’s demographic and socioeconomic status are important determinants of SMM. The incidence of SMM varies according to race and medical coverage [11]. Unstable employment [12], low income [13], and poor living conditions [14] are also associated with SMM.

Recent studies have reported that when hospitals exceed their capacity for delivering babies, the risk of SMM for mothers increases [15]. Furthermore, cohort studies have verified that the incidence of postpartum complications and the risk of SMM can differ based on the time of delivery [16], including nighttime deliveries [17]. However, few large-scale and nationwide cohort studies have investigated the effects of off-hours deliveries, including deliveries on weekends or holidays, on the incidence of SMM.

Therefore, this study evaluated the association between off-hours deliveries and the risk of SMM during hospitalization. The medical environment in Korea (the decline in the demand for and supply of obstetric care due to the rapid decline in fertility) was comprehensively considered to examine whether the effect size differs based on hospital region and bed capacity.

## 2. Materials and Methods

### 2.1. Data and Study Population

This population-based cohort study used a customized cohort dataset provided by the Korean National Health Insurance Service (NHIS). Since the implementation of universal health coverage in 1989, all Korean patients are mandated to be enrolled in the NHIS, and approximately 98% of the population has been enrolled. The NHIS database includes claim data for all cases of Korean medical use, sociodemographic characteristics, diagnosis codes, and drug prescriptions according to the clinically determined International Classification of Diseases tenth revision (ICD-10) codes [18].

The NHIS provides customized cohort data for academic research and policymaking. The delivery cohort used for this study included information regarding medical utilization, sociodemographic characteristics, health status, and death of all pregnant women who delivered in Korean medical institutions between 1 January 2005 and 31 December 2019. Delivery was defined as hospital admission records including pregnancy-related diagnosis or procedure codes for either vaginal or cesarean section delivery among all nulliparous women [19]. A total of 3,076,448 nulliparous women were included in the study population after excluding women with no delivery records at Korean medical institutions and those with missing data.

This study was conducted in accordance with the Declaration of Helsinki and approved by the Institutional Review Board of Eulji University (IRB number: EU22-27). Due to the retrospective nature of the study and the lack of identifiable information in the data, the requirement of informed consent was waived.

### 2.2. Variables

The dependent variable, SMM, was determined using an algorithm developed by United States Centers for Disease Control and Prevention researchers [20]. SMM was defined as the occurrence of at least one of 21 indicators during hospitalization for delivery, including 16 diagnosis codes and five procedure codes representing serious complications related to pregnancy or delivery and procedures performed for the management of such conditions [21,22]. In addition, admission to an intensive care unit or death during the patient’s delivery hospitalization was considered SMM [23].

The main variables of interest were the day and time of delivery, which were identified using Electronic Data Interchange codes [24]. Deliveries were divided into those that occurred during off-hours and those that occurred during office hours. Deliveries that occurred during office hours were categorized as ‘weekday and day’, and those that occurred during off-hours were categorized as ‘weekend and night’, ‘weekend and day’, and ‘weekday and night’.

Sociodemographic factors, obstetric factors, and characteristics of the delivery institution were included as covariates. Sociodemographic factors included maternal age (ranges: 15–19 years, 20–24 years, 25–29 years, 30–34 years, 35–39 years, 40–44 years, and ≥45 years), income level (divided into quartiles: 1Q (lowest) to 4Q (highest)), type of insurance (self-employed, employed, and medical aid), and residential areas (Seoul, metropolitan areas, small cities, and rural). Obstetric factors included the mode of delivery (spontaneous vaginal, instrumental, and cesarean section), preterm birth (yes (<37 weeks’ gestation) and no (≥37 weeks’ gestation)), adequacy of prenatal care (adequate, intermediate, and inadequate), number of fetuses (single and multiple), and maternal comorbidities (yes (≥1) and no (0)). Adequate prenatal care was evaluated according to the Kessner Adequacy of Prenatal Care Index [25], and maternal comorbidities were calculated based on a previous study [26]. In addition, the characteristics of the delivery institution were corrected as potential confounders, including the type of hospital according to the number of beds (<30 beds, 30–99 beds, 100–499 beds, and >500 beds) [24] and the region where the hospital is located (Seoul, metropolitan areas, small cities, and rural). Finally, the year of delivery (2005–2019) was corrected in the analysis.

### 2.3. Statistical Analysis

Descriptive statistics of the general characteristics of the study population are presented as frequencies and percentages. A modified Poisson regression analysis with a sandwich error estimation was conducted to investigate the association between off-hour deliveries and SMM while considering stratification based on hospital region and bed capacity. Adjusted risk ratios (aRR) and 95% confidence intervals (CIs) were calculated. SAS software (version 9.4; SAS Institute Inc., Cary, NC, USA) was used for all analyses. Statistical significance was set at *p* < 0.05.

## 3. Results

The distribution of the study population is presented in Table 1. Among 3,076,448 nulliparous women, SMM occurred in approximately 2074 (2.6%). Approximately 67.3% of mothers gave birth during regular office hours, with 2.5% experiencing SMM. Notably, the incidence of SMM among mothers who delivered during the night (weekday and weekend) was relatively higher at 3.2%. Among the mothers who delivered during the day on a weekend, 2.3% developed SMM.

Table 2 shows the association between off-hour deliveries and SMM after adjusting for the covariates. The probability of SMM was 1.4-fold higher in women who delivered on weekdays and nights (95% CI: 1.38–1.44) and 1.4-fold higher in women who delivered on weekends and nights (95% CI: 1.34–1.46) compared to those who delivered during office hours. Deliveries during the day on the weekend were also associated with an increased risk of SMM (aRR: 1.02, 95% CI: 1.00–1.04).

Deliveries that occurred in hospitals located in small cities had a higher risk of SMM when the delivery took place on weekend nights and weekday nights (weekend and night, aRR: 1.49, 95% CI: 1.40–1.59; weekday and night, aRR: 1.46, 95% CI: 1.42–1.51) (Table 3). The highest risk of SMM was found in women who delivered at night in general hospitals with 100–499 beds (weekend and night, aRR: 1.83, 95% CI: 1.67–2.01; weekday and night, aRR: 1.70, 95% CI: 1.62–1.79).

## 4. Discussion

In this study, we assessed the impact of delivery on the risk of SMM using claims data from all maternity hospitals in Korea between 2005 and 2019. Our findings revealed that off-hour deliveries were associated with a higher probability of SMM than office-hour deliveries, and nighttime deliveries were associated with a greater risk of SMM than weekend deliveries. The extent of the effect of off-hour delivery on SMM risk appeared to be influenced by the availability of obstetric resources, as represented by hospital location and bed capacity.

In this study, the risk of SMM was approximately 1.4 times higher for women who delivered during nighttime hours compared to those who delivered during the weekdays. These results are consistent with those of previous studies investigating the adverse effects of nighttime delivery on perinatal outcomes. A positive association between nighttime birth and SMM has been reported [16], and evening and night delivery is associated with increased perinatal mortality [27]. In addition, the possibility of maternal and neonatal complications increases when delivery occurs on days with a higher-than-usual labor volume or on weekends [28].

The risk of SMM was notably higher when delivery was performed during off-hours in small cities. These findings can be attributed to Korea’s extremely low birth rate and the subsequent decline in obstetric medical resources. As of 2021, the average total fertility rate (TFR) of the Organization for Economic Cooperation and Development countries was 1.63, while Korea’s TFR was only 0.81, resulting in Korea’s classification as an ultralow-fertility country [29]. As the demand for childbirth in Korea decreases, the supply of obstetric care also decreases. With a declining number of obstetricians, there are fewer maternity hospitals and beds in small towns characterized by low birth rates. The results of this study clearly demonstrate the detrimental impact of extremely low fertility rates on maternal health outcomes. Relatively small cities experience a lower demand for deliveries, resulting in an insufficiency of obstetric medical resources. As a result, hospitals may be performing more deliveries than they can handle, compromising the quality of obstetric care and ultimately affecting maternal health.

In addition, it was confirmed that the risk of SMM was particularly high when deliveries occurred during off-hours in general hospitals with 100 to 499 beds. Meanwhile, according to the results described in Table 2, the risk of SMM was highest when delivering in hospitals with more than 500 beds. This discrepancy can be explained by high-risk mothers seeking out hospitals with abundant medical resources. In fact, the impact of off-hour deliveries on the risk of SMM in tertiary general hospitals with more than 500 beds was not as significant as presented in Table 3. In other words, hospitals with abundant medical resources are managing their performance volume well, especially in response to the influx of seriously ill patients. In contrast, most hospitals in small cities capable of caring for seriously ill pregnant women have between 100 and 499 beds, resulting in a concentration of patients in this limited number of general hospitals. This overcrowding phenomenon is more noticeable during off-hours, resulting in a particularly heightened risk of SMM occurrences.

These findings suggest that implementing policies that address the negative impact of low fertility rates and medical inequality on maternal health outcomes is necessary. Allocating obstetric resources to medically vulnerable areas and improving the quality of medical care are essential steps to breaking this vicious cycle. Considering hospital region and type in the Korean medical landscape, our stratified analysis provides valuable insights for the development of effective public health policies. One of the notable strengths of this study lies in its large-scale, nationally representative design, as it includes all mothers who gave birth at medical institutions in Korea between 2005 and 2019. Furthermore, the study utilized SMM, a reliable indicator for measuring maternal mortality rates and overall maternal health conditions [30,31], as the outcome variable. The incidence of SMM in the entire study sample was 2.6%, which is similar to that in previous studies [26,32]. Although one study evaluated the effect of off-hours delivery on SMM using an NHIS cohort database of one million patients [24], the current study is the first to target all of Korea’s parturients and included the most recent periods when the birth rates were decreased.

This study is not without limitations. First, the data used for the study were based on administrative data (including ICD-10 codes) provided by the NHIS, which made it impossible to determine the severity of SMM. Therefore, published algorithms were used to define SMM. Patients’ medical charts were not available to identify patients with SMM. Second, while the data were adjusted for several risk factors that may impact SMM, the possibility of residual confounding effects from unmeasured variables remains. For instance, factors such as body mass index [33], as well as unhealthy habits, such as alcohol consumption [34] and smoking [35], have been identified as risk factors for maternal and child health outcomes but were not included in the current study.

## 5. Conclusions

This study determined the effects of off-hour deliveries on the risk of SMM. It is evident that nighttime delivery poses a more substantial risk for SMM than weekend deliveries. The study also revealed that off-hour deliveries and hospitals in small cities or general hospitals with 100–499 beds were significantly associated with SMM. These results can be attributed to the low birth rate in Korea, which has led to a decrease in obstetric demand and supply in non-urban areas. Consequently, it is imperative that policies that promote maternal health by improving the quality of medical care through the equitable distribution of medical resources, including obstetricians and hospitals, to medically vulnerable areas are developed and implemented.

## Figures and Tables

**Table 1:** Distribution of study population.

Variables		Type of Off-Hours Delivery								Total	
		Weekend and Night		Weekend and Day		Weekday and Night		Weekday and Day			
Severe maternal morbidity											
	No	62,753	96.8	605,177	97.7	312,121	96.9	2,018,005	97.5	2,998,056	97.5
	Yes	2074	3.2	14,526	2.3	10,145	3.2	51,647	2.5	78,392	2.6
Maternal age (years)											
	15–19	599	0.9	3842	0.6	2817	0.9	11,896	0.6	19,154	0.6
	20–24	4885	7.5	42,791	6.9	24,536	7.6	136,045	6.6	208,257	6.8
	25–29	21,182	32.7	215,693	34.8	106,452	33.0	700,507	33.9	1,043,834	33.9
	30–34	31,085	48.0	274,351	44.3	154,323	47.9	908,657	43.9	1,368,416	44.5
	35–39	6437	9.9	73,970	11.9	31,199	9.7	273,501	13.2	385,107	12.5
	40–44	626	1.0	8785	1.4	2867	0.9	37,756	1.8	50,034	1.6
	45+	13	0.0	271	0.0	72	0.0	1290	0.1	1646	0.1
Income level											
	1Q	13,317	20.5	128,950	20.8	66,494	20.6	433,721	21.0	642,482	20.9
	2Q	17,435	26.9	170,476	27.5	88,255	27.4	570,879	27.6	847,045	27.5
	3Q	22,731	35.1	216,009	34.9	112,323	34.9	717,134	34.7	1,068,197	34.7
	4Q	11,344	17.5	104,268	16.8	55,194	17.1	347,918	16.8	518,724	16.9
Type of insurance											
	Self-employed	13,565	20.9	143,863	23.2	69,371	21.5	499,221	24.1	726,020	23.6
	Employed	50,866	78.5	473,310	76.4	250,882	77.9	1,561,966	75.5	2,337,024	76.0
	Medical aid	396	0.6	2530	0.4	2013	0.6	8465	0.4	13,404	0.4
Residential area											
	Seoul	14,401	22.2	141,895	22.9	70,354	21.8	465,656	22.5	692,306	22.5
	Metropolitan areas	16,459	25.4	157,264	25.4	80,518	25.0	512,974	24.8	767,215	24.9
	Small cities	30,186	46.6	284,516	45.9	152,064	47.2	972,173	47.0	1,438,939	46.8
	Rural	3781	5.8	36,028	5.8	19,330	6.0	118,849	5.7	177,988	5.8
Mode of delivery											
	Spontaneous vaginal delivery	30,039	46.3	220,886	35.6	131,941	40.9	507,667	24.5	890,533	29.0
	Instrumental delivery	24,170	37.3	189,517	30.6	135,800	42.1	632,798	30.6	982,285	31.9
	Cesarean section delivery	10,618	16.4	209,300	33.8	54,525	16.9	929,187	44.9	1,203,630	39.1
Preterm birth											
	No	62,635	96.6	603,307	97.4	312,359	96.9	2,019,038	97.6	2,997,339	97.4
	Yes	2192	3.4	16,396	2.7	9907	3.1	50,614	2.5	79,109	2.6
Adequate prenatal care											
	Adequate	61,364	94.7	570,267	92.0	305,255	94.7	1,909,989	92.3	2,846,875	92.5
	Intermediate	3204	4.9	46,111	7.4	15,677	4.9	148,820	7.2	213,812	7.0
	Inadequate	259	0.4	3325	0.5	1334	0.4	10,843	0.5	15,761	0.5
Number of fetuses											
	Single	64,020	98.8	608,782	98.2	318,897	99.0	2,017,941	97.5	3,009,640	97.8
	Multiple	807	1.2	10,921	1.8	3369	1.1	51,711	2.5	66,808	2.2
Maternal comorbidities											
	No	34,095	52.6	358,435	57.8	170,782	53.0	1,176,411	56.8	1,739,723	56.6
	Yes	30,732	47.4	261,268	42.2	151,484	47.0	893,241	43.2	1,336,725	43.5
Type of hospitals (number of beds)											
	>500	5375	8.3	37,555	6.1	23,477	7.3	125,806	6.1	192,213	6.3
	100–499	6593	10.2	65,070	10.5	30,466	9.5	215,006	10.4	317,135	10.3
	30–99	27,187	41.9	270,016	43.6	138,173	42.9	895,744	43.3	1,331,120	43.3
	<30	25,672	39.6	247,062	39.9	130,150	40.4	833,096	40.3	1,235,980	40.2
Hospital region											
	Seoul	14,706	22.7	142,695	23.0	70,583	21.9	471,258	22.8	699,242	22.7
	Metropolitan areas	19,048	29.4	179,187	28.9	92,412	28.7	575,610	27.8	866,257	28.2
	Small cities	30,827	47.6	294,521	47.5	158,107	49.1	1,010,360	48.8	1,493,815	48.6
	Rural	246	0.4	3300	0.5	1164	0.4	12,424	0.6	17,134	0.6
Year											
	2005	1170	1.8	43,883	7.1	6139	1.9	146,192	7.1	197,384	6.4
	2006	1224	1.9	46,217	7.5	6421	2.0	154,261	7.5	208,123	6.8
	2007	1395	2.2	53,095	8.6	7191	2.2	178,038	8.6	239,719	7.8
	2008	1276	2.0	49,447	8.0	6688	2.1	169,373	8.2	226,784	7.4
	2009	1192	1.8	46,952	7.6	6629	2.1	161,414	7.8	216,187	7.0
	2010	5609	8.7	45,095	7.3	28,194	8.8	144,685	7.0	223,583	7.3
	2011	9751	15.0	42,158	6.8	47,391	14.7	128,213	6.2	227,513	7.4
	2012	9414	14.5	44,103	7.1	46,137	14.3	136,376	6.6	236,030	7.7
	2013	7770	12.0	39,587	6.4	37,779	11.7	127,012	6.1	212,148	6.9
	2014	7329	11.3	39,120	6.3	36,171	11.2	125,811	6.1	208,431	6.8
	2015	6964	10.7	39,518	6.4	35,608	11.1	129,098	6.2	211,188	6.9
	2016	5896	9.1	37,096	6.0	28,630	8.9	125,167	6.1	196,789	6.4
	2017	2342	3.6	35,134	5.7	11,757	3.7	125,124	6.1	174,357	5.7
	2018	1924	3.0	31,619	5.1	9755	3.0	115,869	5.6	159,167	5.2
	2019	1571	2.4	26,679	4.3	7776	2.4	103,019	5.0	139,045	4.5

**Table 2:** The association between off-hours delivery and severe maternal morbidity adjusted for all covariates.

	Severe Maternal Morbidity		
	aRR	95% CI	
Off-hours delivery			
Weekend and night	1.40	1.34	1.46
Weekend and day	1.02	1.00	1.04
Weekday and night	1.41	1.38	1.44
Weekday and day	1.00		
Maternal age (years)			
15–19	1.31	1.21	1.43
20–24	1.09	1.05	1.12
25–29	1.00		
30–34	1.13	1.11	1.15
35–39	1.33	1.30	1.36
40–44	1.47	1.41	1.54
45+	1.28	1.04	1.58
Income level			
1Q	1.08	1.06	1.11
2Q	1.07	1.05	1.10
3Q	1.05	1.03	1.07
4Q	1.00		
Type of insurance			
Self-employed	1.09	1.07	1.11
Employed	1.00		
Medical aid	1.57	1.45	1.70
Residential area			
Seoul	1.00		
Metropolitan areas	1.09	1.06	1.12
Small cities	1.16	1.13	1.19
Rural	1.20	1.16	1.24
Mode of delivery			
Spontaneous vaginal delivery	1.00		
Instrumental delivery	1.46	1.43	1.50
Cesarean section delivery	1.92	1.88	1.96
Preterm birth			
No	1.00		
Yes	1.32	1.29	1.36
Adequate prenatal care			
Adequate	1.00		
Intermediate	1.18	1.15	1.21
Inadequate	1.37	1.26	1.50
Number of fetuses			
Single	1.00		
Multiple	1.79	1.75	1.84
Maternal comorbidities			
No	1.00		
Yes	1.44	1.41	1.46
Type of hospitals (number of beds)			
>500	4.65	4.55	4.76
100–499	2.79	2.73	2.85
30–99	1.00		
<30	1.08	1.06	1.10
Hospital region			
Seoul	1.00		
Metropolitan areas	1.28	1.25	1.32
Small cities	1.21	1.18	1.24
Rural	1.25	1.13	1.38
Year			
2005	1.46	1.40	1.53
2006	1.38	1.32	1.44
2007	1.33	1.27	1.39
2008	1.30	1.24	1.36
2009	1.46	1.39	1.52
2010	1.54	1.48	1.61
2011	1.40	1.34	1.46
2012	1.36	1.30	1.42
2013	1.14	1.09	1.19
2014	1.12	1.07	1.17
2015	1.17	1.12	1.22
2016	1.14	1.09	1.19
2017	1.09	1.04	1.14
2018	1.11	1.06	1.17
2019	1.00		

Abbreviations: aRR, adjusted risk ratio; CI, confidence interval. Risk ratios were adjusted for the remaining covariates.

**Table 3:** Subgroup analyses for the association between off-hours delivery and severe maternal morbidity by the hospital region and the number of beds.

		Type of Off-Hours Delivery											
		Weekend and Night			Weekend and Day			Weekday and Night			Weekday and Day		
		aRR	95% CI		aRR		95% CI	aRR	95% CI		aRR		95% CI
Hospital Region													
	Seoul	1.33	1.22	1.45	1.03	0.99	1.06	1.35	1.29	1.41	1.00		
	Metropolitan areas	1.28	1.18	1.40	1.04	1.00	1.07	1.33	1.28	1.39	1.00		
	Small cities	1.49	1.40	1.59	1.00	0.97	1.03	1.46	1.42	1.51	1.00		
	Rural areas	1.34	0.63	2.85	0.82	0.62	1.08	1.84	1.36	2.49	1.00		
Type of hospitals (number of beds)													
	>500	1.19	1.09	1.30	1.05	1.01	1.09	1.19	1.14	1.25	1.00		
	100–499	1.83	1.67	2.01	1.04	0.99	1.08	1.70	1.62	1.79	1.00		
	30–99	1.13	1.03	1.23	1.00	0.97	1.04	1.17	1.12	1.22	1.00		
	<30	1.25	1.14	1.36	0.96	0.93	1.00	1.30	1.24	1.35	1.00		

Abbreviations: aRRs, adjusted risk ratio; CI, confidence interval. Risk ratios were adjusted for the remaining covariates.

## Data Availability

The datasets generated during and/or analyzed during the current study are available from the corresponding author on reasonable request.
